# Single-cell transcriptome profiling reveals intratumoural heterogeneity and malignant progression in retinoblastoma

**DOI:** 10.1038/s41419-021-04390-4

**Published:** 2021-11-23

**Authors:** Jie Yang, Yongyun Li, Yanping Han, Yiyi Feng, Min Zhou, Chunyan Zong, Xiaoyu He, Renbing Jia, Xiaofang Xu, Jiayan Fan

**Affiliations:** 1 Department of Ophthalmology, Ninth People’s Hospital, Shanghai JiaoTong University School of Medicine, Shanghai, P. R. China; 2 Shanghai Key Laboratory of Orbital Diseases and Ocular Oncology, Shanghai, P. R. China

**Keywords:** Eye cancer, Differentiation, Prognostic markers

## Abstract

Retinoblastoma is a childhood retinal tumour that is the most common primary malignant intraocular tumour. However, it has been challenging to identify the cell types associated with genetic complexity. Here, we performed single-cell RNA sequencing on 14,739 cells from two retinoblastoma samples to delineate the heterogeneity and the underlying mechanism of retinoblastoma progression. Using a multiresolution network-based analysis, we identified two major cell types in human retinoblastoma. Cell trajectory analysis yielded a total of 5 cell states organized into two main branches, and the cell cycle-associated cone precursors were the cells of origin of retinoblastoma that were required for initiating the differentiation and malignancy process of retinoblastoma. Tumour cells differentiation reprogramming trajectory analysis revealed that cell-type components of multiple tumour-related pathways and predominantly expressed *UBE2C* were associated with an activation state in the malignant progression of the tumour, providing a potential novel “switch gene” marker during early critical stages in human retinoblastoma development. Thus, our findings improve our current understanding of the mechanism of retinoblastoma progression and are potentially valuable in providing novel prognostic markers for retinoblastoma.

## Introduction

Retinoblastoma is the most common ocular tumour of childhood and is fatal if left untreated. This malignancy is generally detected in infants or young children under the age of 3 years, and 7–10% of retinoblastomas are diagnosed at the neonatal stage during the first month of life and occasionally at birth [1]. Leucocoria is the most common initial sign of retinoblastoma. The management of retinoblastoma is complex and involves strategically chosen methods of enucleation, radiotherapy, chemotherapy, laser photocoagulation, cryotherapy, and thermotherapy [2]. Mortality from retinoblastoma is ~70% in countries of low and middle income and 95–97% in developing countries [3]. Retinoblastoma is thought to result from the inactivation of the *RB1* gene [4]. Studies suggest that biallelic *RB1* inactivation leads to a non-proliferative retinoma, and progression to retinoblastoma requires additional genetic aberrations [5]. However, the cell type in which *RB1* suppresses retinoblastoma and the circuitry that underlies the need for retinoblastoma are undefined. Furthermore, ~2% of retinoblastomas do not harbour *RB1* alterations, and the presence of genetic alterations beyond RB1 inactivation correlates with aggressive histopathologic features [6].

The two-hit hypothesis states that the development of any retinoblastoma requires two complementary tumour-inducing events to convert a normal retinal cell into a neoplastic cell [7]. The debate over the cell of origin of human retinoblastoma has lasted for more than a century. There was evidence that retinal progenitor cells (RPCs) and the inner neuroblastic layer (INL), where bipolar, horizontal and müller transition cells were located, are the cell origins of retinoblastoma [8–10]. Although a recent study showed that cone precursors were the most likely origin, as they had an intrinsic circuitry [11], tumours arising from macula that were rich in cones were fewest in number [12]. The transcriptome of human retinoblastoma had been reported using bulk tissue RNA-seq [13, 14]. These studies provided general transcriptomic information on retinoblastoma as a whole tissue, but the heterogeneity in retinoblastoma and developmental lineages of tumour cells were still unknown.

In recent studies, single-cell separation and sequencing technology made it possible to comprehensively profile the human retina [15–18]. Furthermore, this technology had been applied to identify unrecognized diversity of cell types in uveal melanoma [19] and provided new insights into age-related macular degeneration [20]. Here, for the first time, we captured molecular profiles for human retinoblastoma, indicating the cone precursors and retinoblastoma cells differentiation state in which the highly expressed *UBE2C* gene might serve as an indicator for evaluating the mature and malignancy of retinoblastoma. Our findings provide insight into the developmental trajectories and cellular states underlying human initiation and progression of retinoblastoma.

## Results

### Single-cell RNA sequencing analysis of retinoblastoma

**Fig. 1:**
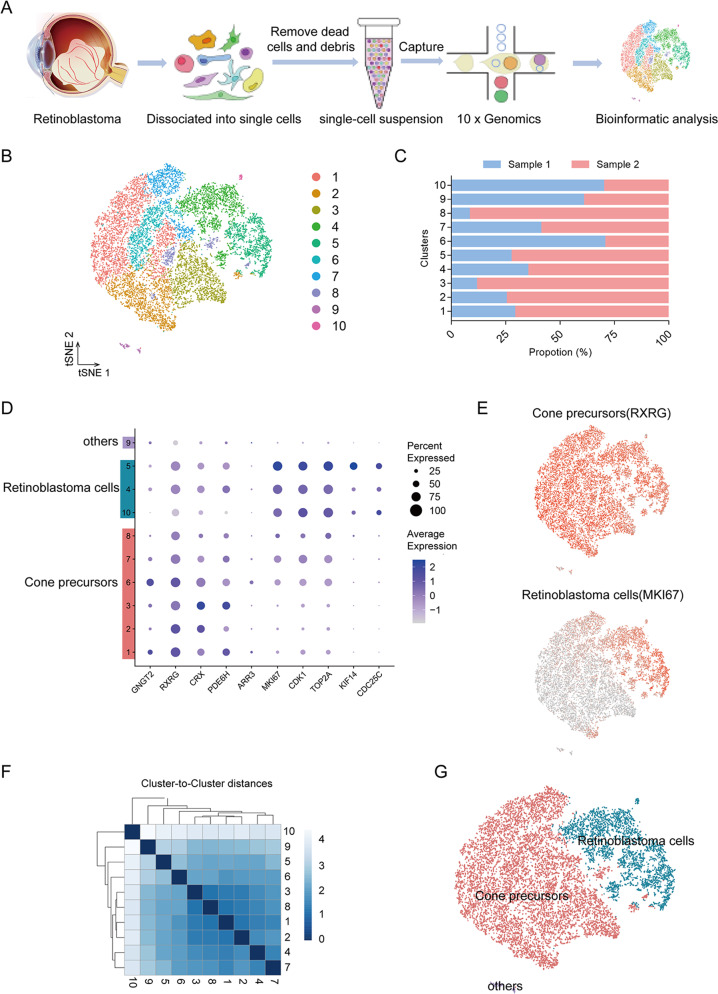
scRNA-seq analysis reveals cellular heterogeneity. **A** Scheme of cell isolation, cell processing, capture by droplet-based device, sequencing, and downstream analysis. **B** t-SNE plot of single cells distributed by unbiased hierarchical clustering. **C** Fraction of cells in each cell cluster. **D** Feature expression heatmap showing expression patterns of major retinal class markers across 10 cell clusters. In a given cell identify, the sizes of circles indicate percentage of the cells expressing each marker gene; The shades of blue indicate average expression of each gene. **E** t-SNE plots showing expression of a set of selected marker genes for major retinal classes. **F** Correlation matrix for the hierarchical clusters. **G** t-SNE visualization of 14,739 cells profiled, with cells colour-coded according to cell type annotation.

To probe the cell type at single-cell resolution, we performed single-cell RNA sequencing (scRNA-seq) on 14739 single cells from two retinoblastoma tumour samples (Fig. 1A). The patients were all diagnosed as group E advanced retinoblastoma with endophytic type of tumour growth. Enucleation was the primary treatment without any other treatments (Supplementary Fig. 1, Supplementary Table 1). Following preprocessing and quality control (QC) (Supplementary Fig. 2A, B), we obtained high-quality transcriptomic data from a total of 14739 cells. The majority of the sequenced cells had 2991-4172 genes and 9709-16818 median unique molecular identifiers (UMIs) associated with the cell barcodes (Supplementary Fig. 2C). After QC, the scRNA-seq data were initially analysed using an unsupervised graph clustering approach implemented in Seurat to classify individual cells into cell populations according to similarities in their transcriptome profiles. Overall, the cells were classified into 10 transcriptionally distinct clusters using a t-distributed stochastic neighbour embedding (t-SNE) plot, where each dot represented a single cell (Fig. 1B). Each cluster consisted of cells in the range of 53-3610. The proportion of cells in each cluster was shown in Fig. 1C, suggesting relatively low sample bias. Notably, unsupervised clustering and t-SNE analysis showed the cluster 9 (c9) that was transcriptionally distinct from all other clusters. These results indicated high intratumoural heterogeneity in retinoblastoma.

To identify major cell types in human retinoblastoma, cells-specific genes were used to annotate cell types with classic markers described in previous studies [11, 21]: cone precursors (*GNGT2, RXRG, CRX, PDE6H*) and retinoblastoma cells (*MKI67, CDK1, TOP2A, KIF14, CDC25C*). We then generated cluster-specific marker genes by performing differential gene expression analysis to define the identity of each cell cluster (Fig. 1D). In most cases, well-known cell type markers were used to identify cell clusters, such as *RXRG* for cone precursors [11], *MKI67* for retinoblastoma cells [21] (Fig. 1E). As expected, we observed high correlations between the expression levels of transcripts within the same cell type (Fig. 1F). We also identified multiple additional other retinal cell types markers [15, 17, 20, 22, 23], such as rods (*PDE6A, RHO, NR2E3*), mature cones (*OPN1LW, OPN1MW, OPN1SW*), Müller glia (*RLBP1, APOE, CLU*), retinal astrocytes (GFAP), microglia (*HLA-DPA1, HLA-DRA, C1QA*), bipolar cells (*VSX2, TMEM215, VSX1*), retinal ganglion cells (*SNCG, SLC17A6, RBPMS*), amacrine cells (*CALB1, CHAT, GAD2*), and horizontal cells (*ONECUT1, PROX1, LHX1*) (Supplementary Fig. 3A). These genes were barely expressed. We observed c9 expressed markers from multiple retinal cell types (Supplementary Fig. 3B). Thus, we were unable to assign cell identities to the cluster and it was excluded from further analysis. Therefore, our data demonstrated there were at least two types of cells in retinoblastoma. The main cell types were cone precursors (six clusters: c1-3, and 6-8) and retinoblastoma cells (three clusters: c4, 5 and 10) with multiple transcriptionally distinct clusters (Fig. 1G).

### Cell cycle-associated cone precursor is the cell of origin of retinoblastoma

**Fig. 2:**
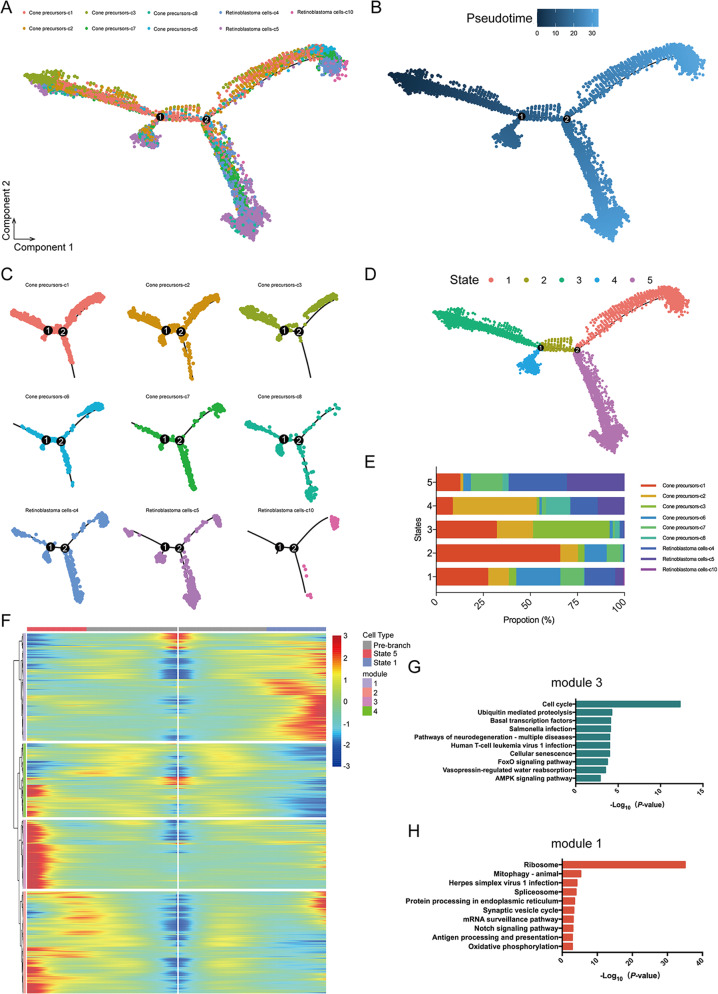
Construction of cone precursors and retinoblastoma cells differentiation trajectories. **A**, **B** Trajectory analysis revealing the retinoblastoma progression, colour-coded according to cell clusters (all six subtypes of cone precursors and three subtypes of retinoblastoma cells). **C** Pseudotime trajectory analysis of individual clusters (c1, c2, c3, c4, c5, c6, c7, c8, c10). **D** The trajectory showed state-specific progression patterns in retinoblastoma. **E** Composition of the clusters within each cell state. **F** Heatmap showing the ordering gene expression dynamics during the cellular-state transition process and expression dynamics. State-1 and state-5 reprogramming trajectories (including pre-branch) are shown on the right and left, respectively. **G**, **H** Heatmap of KEGG enrichment analysis enrichment by module 3 (**G**) and module 1 (**H**).

It is not yet clear which retinal cell type is the cancerous origin of retinoblastoma. Some studies supported the origin of retinoblastoma from photoreceptor precursor cells [24], while accumulating evidence suggested that retinoblastoma was primarily derived from cone precursors [11, 25]. However, these findings were mainly based on observations from transgenic mouse models and had not been validated in human retinoblastoma. To confirm the cell of origin of retinoblastoma, we performed pseudotime trajectory analysis (Fig. 2A, B). Our data showed that cone precursors and retinoblastoma cells demonstrated a relatively linear developmental progression. Notably, across the developmental trajectory specific to retinoblastoma, two subtypes of cone precursors (c7, 8) were present at the branch point 2, followed two branches by retinoblastoma cells (Fig. 2C). The branches separated the cell trajectory into five states (Fig. 2D, E). To further investigate this ongoing process, we performed RNA velocity analysis to predict the potential direction and speed of cell state transitions. Notably, it ﻿was consistent with ﻿the results of pseudotime trajectory analysis (Supplementary Fig. 4). These results suggested that the cone precursor cells might develop into premalignant cone precursors, transform into retinoblastoma cells in two states.

Next, we analysed the gene expression heatmap of ordering genes in a pseudo-temporal order to elucidate the molecular dynamics that distinguished two branches. ﻿Four major gene modules were identified accounting for the distinctions (Fig. 2F). The data showed the pre-branch (state-3) cell populations at earlier stages and after bifurcation into two branches (state-1 and 5). Obviously, the state-3 cluster initiated the delamination of retinoblastoma, and branch cells in state-5 expressed higher levels of cell cycle-related genes (Fig. 2G). The branch cells in state-1 ﻿expressed higher levels of genes enriched for the KEGG terms “ribosome”, ﻿“mitophagy-animal” and “spliceosome” (Fig. 2H). These results indicated that state-5 cell populations were gradually shifting the malignancy process, which was indicative of their ongoing maturation.

**Fig. 3:**
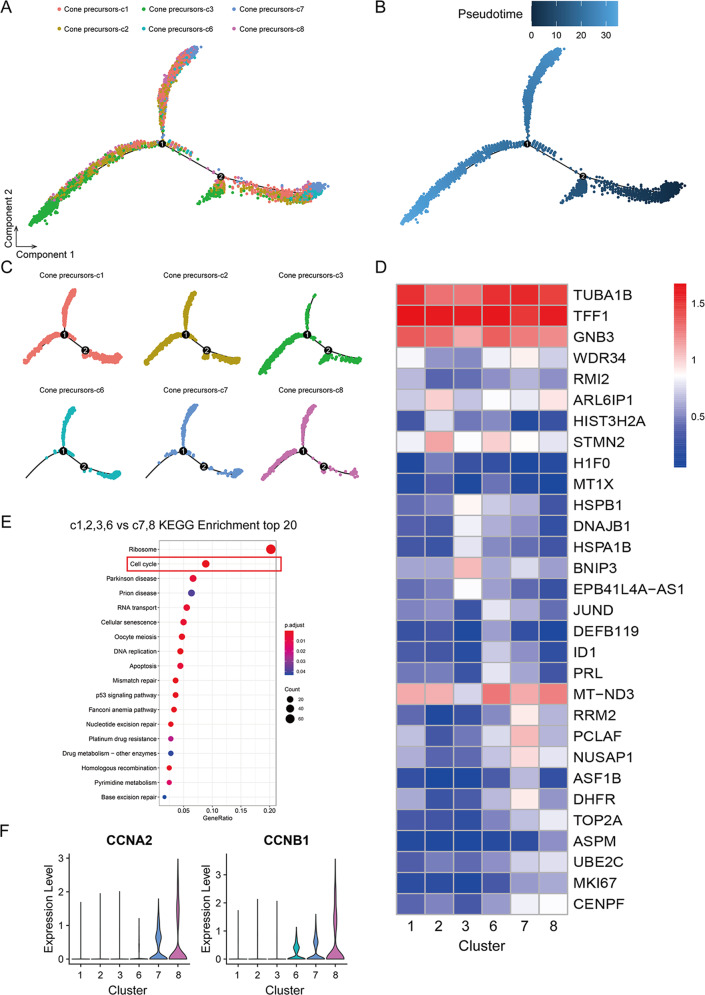
Trajectory analysis of cone precursors. **A**, **B** Trajectory analysis revealing the cone precursors progression, colour-coded according to cell clusters (all six subtypes of cone precursors). **C** Pseudotime trajectory analysis of individual clusters (c1, c2, c3, c6, c7, c8). **D** Heatmap displaying normalized expression of top 5 DEGs among six subtypes of cone precursors (c1, c2, c3, c6, c7, c8). **E** KEGG enrichment analysis identified the top 20 pathways between c1, c2, c3, c6 and c7, c8. **F** Violin plot for the expression of cell cycle genes (CCNA2 and CCNB1) in six subtypes of cone precursors (c1, c2, c3, c6, c7, c8).

To further elucidate the cell of origin, we compared the six clusters of cone precursors. Pseudotime trajectory analysis of cone precursors (Fig. 3A, B) suggested that c7 and c8 were differentiation lately, compared to others cluster (Fig. 3C). As displayed in the heatmap of the average number and distribution of top five differentially expressed genes (DEGs) in each cone precursors cluster, the transcriptome could be distinguished into two subgroups (c1, 2, 3, 6 and c7,8) according to the proportion of highly expressed genes (Fig. 3D). It has been reported that *RB* loss induces cell cycle entry in immature (ARR3−) but not in maturing (ARR3+) cone precursors, as cone precursors were uniquely sensitive to *RB* depletion in retinoblastoma cells [26]. Similar observations were also obtained in our jitter plots analysis which showed that *RB1* and *ARR3* were barely expressed in these cells (Supplementary Fig. 5A, B), suggesting that the six clusters of cone precursors were immature. In contrast, *MYCN* was highly expressed in c5 (retinoblastoma cells) a gene played crucial roles in tumour cell proliferation that was consistent with previous study [25] (Supplementary Fig. 5C). Interestingly, we also observed *ATOH7* was relatively high expression in state-4 particularly in c2 (Supplementary Fig. 5D), a gene promotes cone genesis in human retinal development [23]. These results showed the presence of heterogeneity within cone precursors in retinoblastoma and raised the possibility that cone precursors might still have potential normal differentiation function in retinoblastoma.

Since proliferation was found to be the main feature of retinoblastoma, we then performed functional enrichment analysis for each subgroup and found that the unique functions of subgroup c7, 8 were related to cell cycle (Fig. 3E), which was further supported by the specific expression of cell cycle genes: *CCNA2* and *CCNB1* (Fig. 3F). Taken together, these results showed that the subtypes of cone precursors with high cell cycle-related gene expression were the major origin source of retinoblastoma.

### Identifying malignant programs of retinoblastoma

**Fig. 4:**
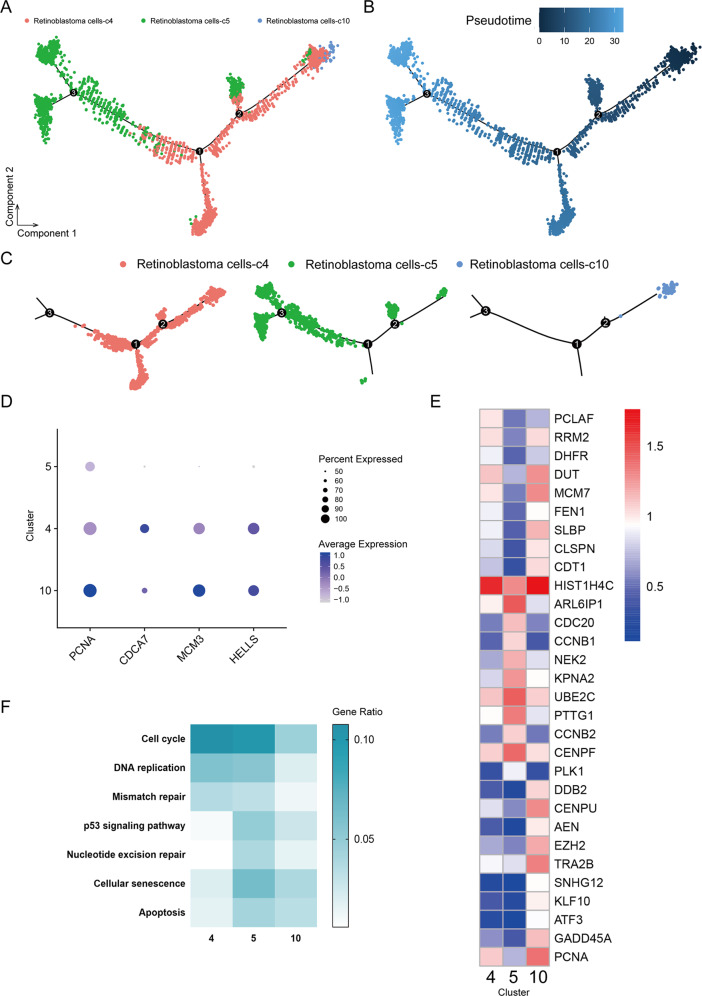
Trajectory analysis of retinoblastoma cells. **A**, **B** Trajectory analysis revealing the retinoblastoma cells progression, colour-coded according to cell clusters (all three subtypes of retinoblastoma cells). **C** Pseudotime trajectory analysis of individual clusters (c4, c5, c10). **D** Feature expression heatmap showing expression patterns of select markers across three retinoblastoma cell clusters. The size of each circle depicts the percentage of cells expressing the marker within the cluster. Purple colour indicated the average expression (number of transcripts). **E** Heatmap displaying normalized expression of top 10 DEGs among three subtypes of retinoblastoma cells (c4, c5, c10). **F** Heatmap of KEGG enrichment analysis enrichment by clusters (c4, c5, c10).

The presence of three subtypes of retinoblastoma cells in tumour prompted us to investigate their malignant status. To define malignant cells, we firstly profiled pseudotime trajectory analysis of retinoblastoma cells (Fig. 4A, B). The three subtypes were inconsistent in transcriptome and gene differentiation (Fig. 4C and Supplementary Table 2). Based on cluster-specific marker genes in previous studies, we observed c10 strongly expressed the markers of retinoma-like cells (PCNA, CDCA7, MCM3, HELLS) [21], decreasing in c4 and barely in c5 (Fig. 4D). Thus, we speculated that retinoma-like cells could be an intermediate cell stage between premalignant cone precursors and tumour cells. Meanwhile, the functional enrichment analysis showed that genes upregulated in c5 cells were mainly enriched for cancer-related functions, such as cell cycle, DNA replication, p53 signalling pathway and apoptosis (Fig. 4E, F).

**Fig. 5:**
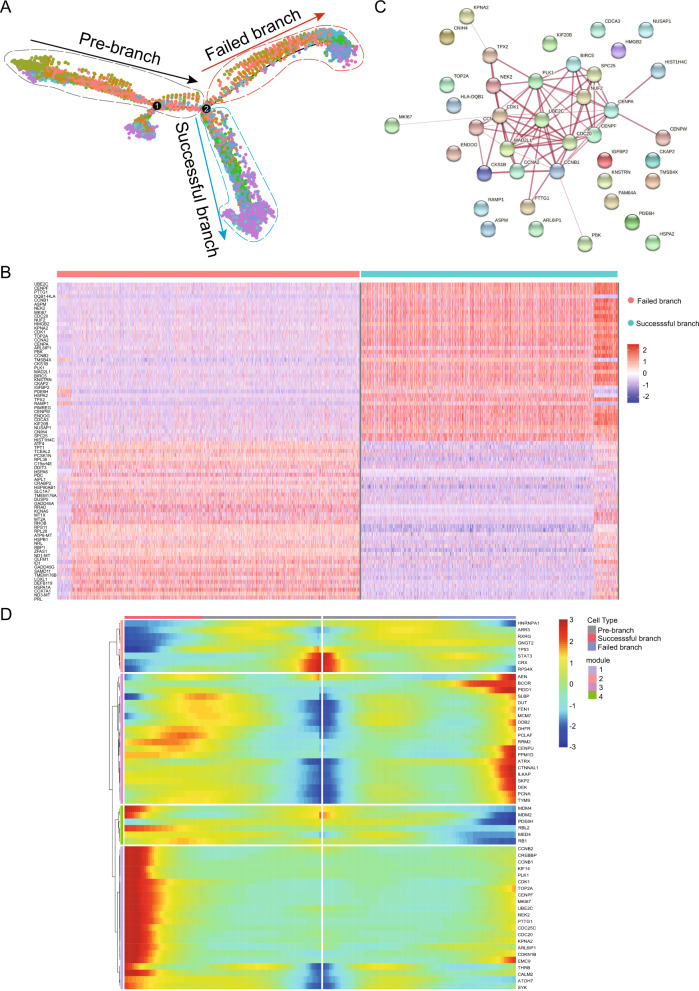
Reconstruction of Reprogramming Trajectory in a Pseudotime Manner. **A** Trajectory reconstruction of all single cells throughout chemical reprogramming reveals three branches: pre-branch (before bifurcation), successful branch, and failed branch (after bifurcation). Blue and red circles indicate cells of the successful and failed branches, respectively. **B** Heatmap showing the top 40 DEGs expression levels in successful branch and failed branch cells. **C** Protein-protein interaction network of the top 40 DEGs in successful branch. **D** Heatmap showing the select genes expression dynamics during the cellular-state transition process and expression dynamics. The successful and failed branches reprogramming trajectories (including pre-branch) are shown on the right and left, respectively.

Liu et al. showed that dynamic expression of genes, such as *SYK*, *DEK* and *NSE* from retinoma-like cells to cancerous organoids was strong in the bridge state [21]. To test this possibility, we firstly characterized the trends of all single cells along pseudotime (Fig. 5A). As expected, we noted that cone precursors were the root cells that were required for initiating the delamination and migration process of retinoblastoma development, while determination of the fate of retinoblastoma cells was accompanied by increased UBE2C expression (Fig. 5B). The cells enriched in state-5 at the terminal of the branch shared a highly similar global expression profile with retinoblastoma cells that possess cell cycle (*UBE2C*, *PTTG1*, *CCNB1*) and proliferation (*MKI67*) properties (Fig. 5B). In addition, *UBE2C* represented as a “pivot” gene in retinoblastoma cells branch (Fig. 5C). Meanwhile, the cells enriched in state-1 at the terminal of the branch maintained high expression profile (*MCM7*, *PCNA*) with retinoma-like cells. Similarly, we also detected consistent results in pseudotime heatmap (Fig. 5D).

This finding suggested that there was reconstruction of malignant tumour cells differentiation reprogramming trajectory during the development of retinoblastoma and *UBE2C* may be a newly proposed oncogene with functions in tumorigenesis.

### *UBE2C* with potential malignant transformation capacity in retinoblastoma

**Fig. 6:**
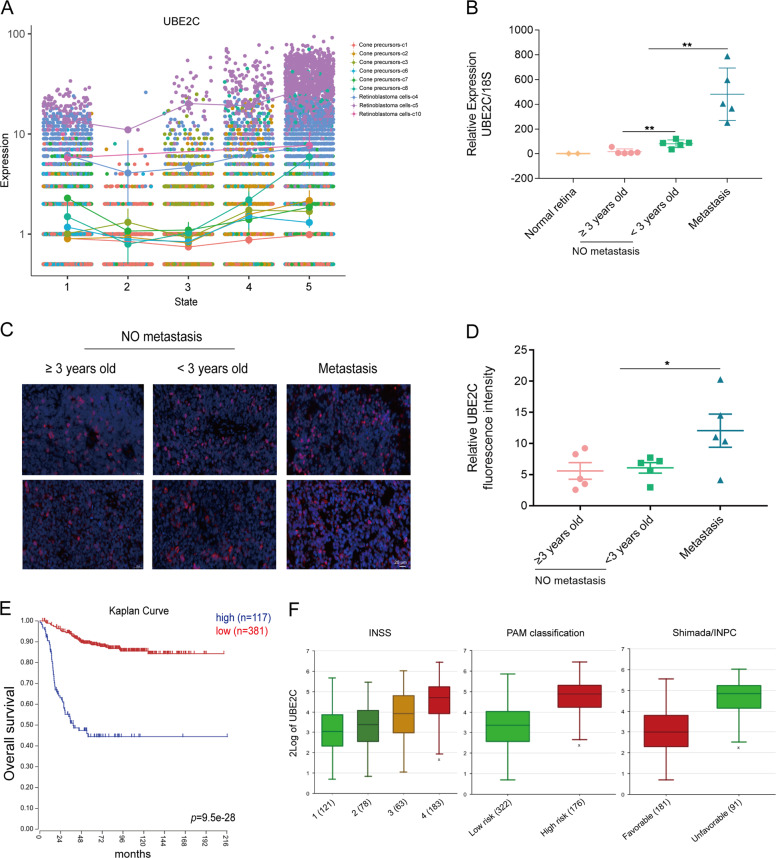
UBE2C is an independent prognostic factor in retinoblastoma. **A** Jitter plots showing the expression level of the UBE2C changing with pseudotime, colour-coded according to cell clusters (c1, c2, c3, c4, c5, c6, c7, c8, c10). **B** Real-time PCR showed UBE2C expression in retinoblastoma and normal tissues. **P < 0.01. **C** IF staining of UBE2C in different age stages and metastasis/nonmetastatic retinoblastoma. Bars: 20 μm. **D** The results of IF analysis of UBE2C expression are presented of the mean number of the UBE2C -positive rate. *P < 0.05. **E** Correlation between UBE2C mRNA expression and neuroblastoma patient survival in the TCGA dataset. Overall patient survival in groups with high and low UBE2C expression was analysed using a Kaplan–Meier survival curve. **F** High-level UBE2C mRNA expression in neuroblastoma correlates with unfavourable clinical features, including high INSS stage, high risk of PAM classification and unfavourable Shimada/INPC in a cohort of 476 tumours.

Indeed, UBE2C expression had already been shown to have the potentially ability to regression of tumours and was a reliable prognostic factor. However, few studies explored the role of the *UBE2C* in retinoblastoma. The results clearly showed that *UBE2C* was remarkably increased in state-5, especially in retinoblastoma cells-c5 (Fig. 6A). To verify the clinical significance of *UBE2C*, we collected a set of tumour tissues paired with normal tissue (GEO accession number: GSE111168). The bulk RNA-seq analysis revealed that all *UBE2C* transcripts were highly expressed (Supplementary Fig. 6A). This finding strongly suggested that *UBE2C* amplification potentiates a progenitor-like proliferative state. To explore the prognostic role of *UBE2C* in retinoblastoma, we then examined the expression of *UBE2C* in bulk tumours. As expected, *UBE2C* presented high expression in tumours and was higher in younger patients (<3 years old) (Fig. 6B). In addition, a prominent increase in UBE2C expression was detected in metastatic patients (Fig. 6B). The results clearly showed that UBE2C protein expression was remarkably increased in the tissues of retinoblastoma corrected from the younger patients and especially the metastasis patients compared with that of children over 3 years old. Similarly, we also detected consistent results in immunofluorescent staining assay (Fig. 6C, D). These findings were consistent with previous clinical observations from Shiedls’ team, which showed that patients with a younger age at diagnosis had a higher genetic risk of developing second malignant neoplasms than older patients at diagnosis.

We next explored the prognostic significance of *UBE2C* in retinoblastoma. However, current studies on retinoblastoma lacked a consistent and measurable database. Fortunately, numbers researches have been indicated that retinoblastoma shared the same mechanisms of tumour formation as another paediatric tumour neuroblastoma [27]. Furthermore, chemotherapy protocols used in treating retinoblastoma closely mimic those used in neuroblastoma management. Thus, here we examined the prognostic role of *UBE2C* in neuroblastoma. ﻿We reanalysed RNA-seq data from a cohort of 498 neuroblastomas [28]. Survival analysis demonstrated that patients with higher expression of UBE2C display significantly lower survival rate, suggesting potential prognostic biomarker (Fig. 6E). In addition, high-level UBE2C expression also significantly correlated with established clinical and molecular markers for unfavourable tumour biology, including INSS stages, a high-risk tumour transcriptional profile defined by principal access method (PAM) analysis and unfavourable Shimada/INPC tumour histology (Fig. 6F). Moreover, UBE2C was significantly upregulated in a series of tumour (Supplementary Fig. 6B), which were positively correlated to pathogenic condition and prognosis (Supplementary Fig. 6C). These data further highlighted the clinical importance of *UBE2C* in tumours.

### Suppression of UBE2C inhibits tumour progression in vitro and in vivo

**Fig. 7:**
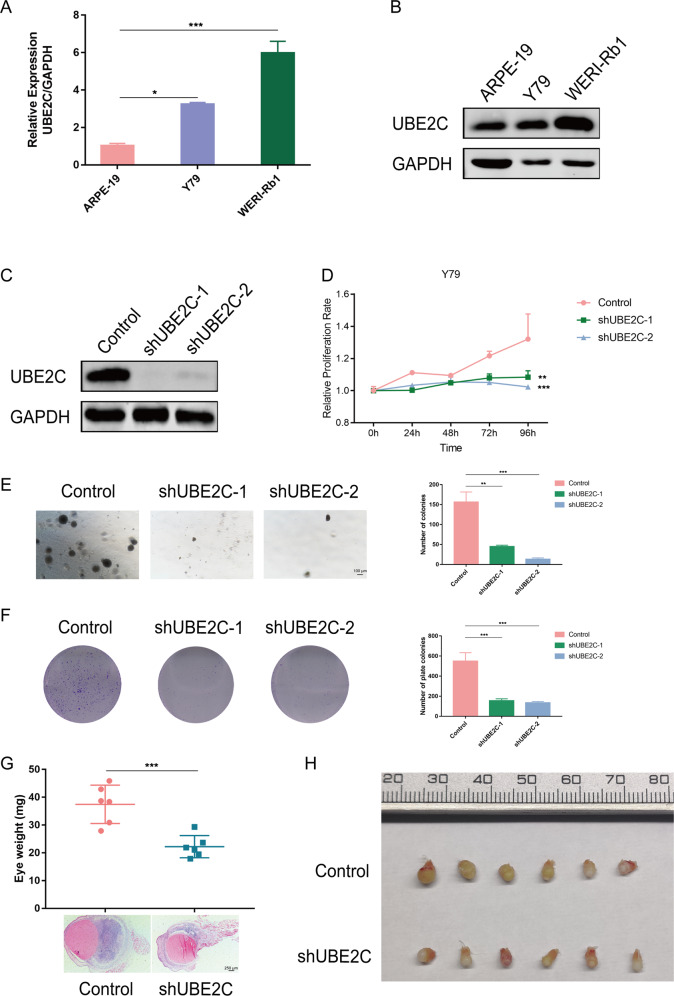
Knockdown of UBE2C inhibits cell proliferation in vitro and in vivo. **A** Real-time PCR was used to evaluate the mRNA level of UBE2C in retinoblastoma cell lines. **B** The protein levels of UBE2C were detected by western blot in retinoblastoma cell lines. **C** Knockdown of UBE2C protein in Y79 cells was assessed by western blot. **D** Knockdown of UBE2C inhibited the proliferation of Y79 cells. **P < 0.01, ***P < 0.001. **E** A soft agar assay was used to assess the tumour formation ability in vitro. Small colonies were observed and counted under the microscope. Colonies were photographed (left panel) and quantified (right panel). Scale bar: 100 μm. **F** A colony formation assay was performed to determine the colony formation influence of UBE2C-knockdown Y79 cells. Colonies were photographed (left panel) and quantified (right panel). **G** Top: Eye weight of the orthotropic xenograft formed by UBE2C-knockdown and Control Y79 cells injected at 30 days after implantation; *n* = 6, ***P < 0.001. Bottom: H&E staining to evaluate tumour formation. Scale bar: 250 μm. **H** Representative image of tumour bearing eyeballs removed from the mice at the 30th day.

To provide evidence for the specificity of *UBE2C* in retinoblastoma, we then examined the expression of UBE2C in retinoblastoma cell lines. As expected, we found that UBE2C was highly expressed in the retinoblastoma cells compared with that in the normal control ARPE-19 cells (Fig. 7A, B). Whether the tumour characteristics were significantly altered after UBE2C knockdown was then investigated. First, we aimed to knockdown the expression of UBE2C by using one control cell line with a mock virus carrying an empty vector. We then detected whether the expression of UBE2C was knocked down in two UBE2C-knockdown Y79 cells by western blot (Fig. 7C). Next, we estimated the role of UBE2C in Y79 cells. In the CCK8 assay, tumour cell growth was significantly decreased in all the UBE2C-knockdown Y79 cells, whereas the control cells retained a higher cell viability (Fig. 7D). Next, we used a classical soft agar assay to examine tumour formation ability in vitro. We also observed that the UBE2C-knockdown cells formed smaller colonies (Fig. 7E). Consistently, in a colony formation assay, the number of colonies of UBE2C-knockdown Y79 cells colonies was significantly reduced (Fig. 7F).

To further investigate the role of UBE2C in vivo, we injected UBE2C-knockdown Y79 cells into subretinal spaces of nude mice to establish orthotopic xenograft models. Compared with Control group, UBE2C-knockdown group had significantly inhibited tumour growth and reduced tumour volumes and weights (Fig. 7G, H).

Taken together, *UBE2C* played a regulatory role in retinoblastoma progression in vitro and in vivo, and could be regarded as a potential therapeutic target.

## Discussion

Retinoblastoma is the most common intraocular tumour of childhood and represents 11% of cancers developing in the first year of life. In some forms of childhood cancer, it has been proposed that synchronous lesions that are in separate anatomical regions may represent independent tumours [29]. Thus, it is highly desirable to explore the intratumoural heterogeneity and the underlying mechanism that are pivotal for retinoblastoma prognostic improvement. In this study, we generated a reference single-cell transcriptome atlas and revealed the retinal cell type-specific components inside retinoblastoma tissues.

We obtained a mean sequencing depth of 55690-102261 reads per cell across 14,739 cells, which enabled us to confidently classify the majority of cell types in complex tumour tissues, such as retinoblastoma. Less transcriptionally distinct cell types mainly included cone precursors and retinoblastoma cells. In addition, the cone precursors had 6 subtypes and retinoblastoma cells had 3 subtypes. However, the ability to resolve these subtypes might be improved by increased sample size, greater cell numbers, or ultradeep sequencing of those populations.

An intriguing common theme has emerged wherein the expression of disease-associated genes was cell-type-specific in the adult retina, and cell-type specificity was retained in organoids [18]. Although the response of retinal cells to the early loss of *RB1* is clearly understood, retinoblastoma cells of origin remain debatable. However, this study provided new insight into retinoblastoma, a childhood retinal tumour, at the single-cell level. Cone precursors and retinoblastoma cells, two major cell types with different transcriptomic profiles, were identified in retinoblastoma. Coincidentally, in 2021, Collin et al. also used single-cell sequencing to verify that ﻿G2/M cone precursors subset was ﻿the cell of origin for retinoblastoma [30]. This was consistent with our conclusion.

Notably, pseudotime trajectory showed five distinct states of tumours. We depicted the trajectory of malignant tumour cells differentiation reprogramming. It was clearly showed that two cone precursors subtypes of c7 and c8 which highly expressed the cell cycle-genes were the cell origin of retinoblastoma. The differentiation trajectory started from the immature cone precursors (pre-branch) (state-3), and was divided into two branches, one branch (state-5) differentiates into mature retinoblastoma cells, and the other (state-1) differentiates into retinoma-like cells. Thus, we speculated that retinoma-like cells could be an intermediate cell stage between premalignant cone precursors and tumour cells. Retinoma or retinocytoma could cause leukocoria and accounts for 3% of pseudo retinoblastomas has redefined as a precancerous lesion characterized by the loss of function of both copies of the *RB1* gene, but lacking the additional genomic changes characteristic of retinoblastoma [31]. There is the evidence that retinoma or retinocytoma is a precursor of retinoblastoma. Rare cases of clinically documented malignant transformation have been reported, and photoreceptor differentiation has been observed repeatedly at the base of endophytic retinoblastomas in enucleated eyes [32, 33].

Multiple stage-specific genes were previously implicated in cone precursors’ capacity to model retinoblastoma initiation, proliferation, premalignant arrest, and tumour growth [26]. The initial expression of *ARR3* coincides with the emergence of cone outer segmented and the appearance of apically positioned concentrated actin filaments that were implicated in outer segment development [26]. Concordantly, *ARR3* was barely detectable in immature cone precursor cells, as a previous study suggested that cone precursor maturation was associated with increased *ARR3* [26]. Incidentally, *ARR3* was initially expressed at the state-2, which was dominated by cone precursors-c1. This tropism further suggested that the immature cone precursors were required for initiating the delamination and migration process of retinoblastoma development. Recently, scRNA-seq analysis revealed that *ATOH7* promoted cone genesis during early critical stages in human retinal development when retinal neurogenesis was initiated [23, 34]. In this report, however, we found *ATOH7* was relatively highly expressed in cone precursors-c2. Highly differentiated neuroblastoma, as estimated by a histology grading system, could undergo spontaneous cellular differentiation or regression without therapy [35]. Clinically recognized retinoblastoma has been found to undergo “spontaneous regression” in <5% of cases [36]. Thus, new spontaneous genetic events of *ATOH7* may contribute potential normal differentiation function in retinoblastoma.

Although we cannot eliminate other genes promoted tumorigenesis during critical stages in retinoblastoma development, this is the first study to imply that *UBE2C* as the crucial transcription regulatory factor during the malignant tumour cells differentiation reprogramming. *UBE2C* encodes a member of the E2 family that guides polyubiquitination to targeted lysine in substrates and plays important roles in the cell cycle and checkpoint control through cyclin B destruction [37, 38]. The *UBE2C* gene was reported to be highly expressed in a variety of solid tumours [39–43] and remains an independent adverse prognostic factor for relapse and death in high-risk breast cancer [44]. However, there was no evidence indicate the regulatory role of the *UBE2C* gene in retinoblastoma. In this report, we clearly demonstrated that *UBE2C* was strongly correlated with the degree of malignancy and metastasis of retinoblastoma. It should be noted that the expression of *RB1* and *UBE2C* was negatively correlated (Supplementary Fig. 7) which suggested that RB1 malfunction might be related to UBE2C overexpression. In eukaryotes, the ubiquitin proteasome system requires the ubiquitin-activating enzyme (E1), the ubiquitin-conjugating enzyme (E2) and ubiquitin ligases (E3) to work in concert to facilitate ubiquitination of target proteins. UBE2C accepts ubiquitin from E1, transfers it to specific anaphase promoting complex/cyclosome (APC/C) E3 complex substrates and catalyses lys-11- and lys-48-specific polyubiquitination, finally contributing to degradation of the APC/C substrates [45, 46]. Thus, *UBE2C* acts as the critical gene that might coordinately regulate the occurrence of intratumoural heterogeneity and further tumour progression in retinoblastoma. It would be of great interest to focus on the identification of other factors to better understand RB1 malfunction.

Our analysis reveals that tumours contain multiple cell states with distinct transcriptional programs and provides inferential evidence for dynamic transitions. A better understanding of the spectrum and dynamics of cellular states in retinoblastoma is thus critical for establishing faithful models and advancing therapeutic strategies that address the complexity of this disease.

## Materials and methods

### Patients and sample collection

Human tissue samples were obtained with patient informed consent and approval of the Shanghai Jiao Tong University research ethics committee. Immediately following surgical eye removal, the tissue was dissected to isolate the tumour region for single-cell dissociation. The normal control retina was a donor from a 2-year-old congenital heart disease.

### Tissue processing for single-cell suspension

Tissue samples were placed immediately in a 50 mL centrifuge tube containing 5 mL of DPBS with 10% FBS. The “Dissociation of soft tumours” protocol from the Miltenyi Tumour Dissociation Kit, human was used with a slight modification. Briefly, samples were incubated at 37 °C for 30 min in a shaker. Samples were passed through a 40 μm cell strainer (Miltenyi Biotec). After the initial incubation step, cells were kept on ice for the remainder of the protocol. The cell suspension then underwent a protocol utilizing Red Blood Cell Lysis Solution (10 X, Miltenyi Biotec) and the Maglive Dead Cell Removal Kit (QDSphere), a density gradient method to remove erythrocytes, dead cells and debris. Samples were processed from surgical removal to loading on the Chromium (10× Genomics) instrument immediately.

### Single-cell RNA sequencing analysis

The Cell Ranger software pipeline (version 3.0.0) provided by 10× Genomics was used to demultiplex cellular barcodes, map reads to the genome and transcriptome using the STAR aligner, and downsample reads as required to generate normalized aggregate data across samples, producing a matrix of gene counts versus cells. We processed the UMI count matrix using the R package Seurat (version 3.1.1) [45]. To remove low-quality cells and likely multiplet captures, which was a major concern in microdroplet-based experiments, we applied criteria to filter out cells with UMI/gene numbers out of the limit of mean value ± 2-fold standard deviations assuming a Gaussian distribution of each cell’s UMI/gene numbers. Following visual inspection of the distribution of cells by the fraction of mitochondrial genes expressed, we further discarded low-quality cells where >10% of the counts belonged to mitochondrial genes. After applying these QC criteria, 14,739 single cells remained and were included in downstream analyses. Library size normalization was performed in Seurat on the filtered matrix to obtain the normalized count.

The top variable genes across single cells were identified using the method described in Macosko et al. [46]. Briefly, the average expression and dispersion were calculated for each gene, and genes were subsequently placed into 10 bins based on expression. Principal component analysis (PCA) was performed to reduce the dimensionality of the log-transformed gene-barcode matrices of the top variable genes. To remove the batch effect affecting downstream analysis, we adopted a method called mutual nearest neighbours (MNN) presented by Haghverdi et al. [47]. Cells were clustered based on a graph-based clustering approach and visualized in two dimensions using t-SNE. A likelihood ratio test that simultaneously tests for changes in mean expression and in the percentage of expressed cells was used to identify significantly DEGs between clusters.

DEGs were identified using the FindMarkers function of the Seurat package [45]. A *P* value <0.05 and |log2fold change| > 0.58 were set as the thresholds for significantly differential expression. KEGG pathway enrichment analyses of DEGs were performed using R based on the hypergeometric distribution.

### Pseudotime trajectory analysis

We determined the developmental pseudotime with the Monocle2 package [48]. The data, previously scaled and clustered by the Seurat tool, were loaded into a monocle object with default parameters. We obtained variable genes with Monocle2 and ordered the cells onto a pseudotime trajectory based on the union of highly variable genes obtained from all cells. Gene expression dynamics underlying cell state transitions could be inferred by ordering the cells based on single-cell expression profiles.

### RNA velocity analysis

To perform the RNA velocity analysis, the spliced reads and unspliced reads were recounted by the velocyto python package based on previously aligned bam files of scRNA-seq data. The calculation of RNA velocity values for each gene in each cell and embedding the RNA velocity vector into low-dimensional space were performed with the R package velocyto.R v0.6 [49]. Velocity fields were projected onto the t-SNE embedding obtained in Seurat and the pseudotime space produced by Monocle 2.

### RNA extraction and reverse transcription-PCR analysis

Total RNA was extracted using TRI-Reagent (Invitrogen, USA), and cDNA was synthesized using the PrimeScript RT reagent kit according to the manufacturer’s instructions (Takara, Japan). Real-time PCR analyses were performed using Power SYBR Green PCR Master Mix (Applied Biosystems, Irvine, CA, USA) on a Roche LightCycler 480 System. The primers were as follows: UBE2C, sense: 5′-GACCTGAGGTATAAGCTCTCGC−3′ and UBE2C, antisense: 5′- CAGGGCAGACCACTTTTCCTT−3′. The relative expression of individual transcripts was normalized to 18S rRNA. The fold change of target mRNA expression was calculated based on the threshold cycle (Ct), where ΔCt = Cttarget−Ct18S and Δ (ΔCt) = ΔCt Control−ΔCt Indicated condition.

### Immunofluorescence assays

The slides were deparaffinized and rehydrated, immersed in sodium citrate antigen retrieval solution (pH 6.0) and blocked with 3% bovine serum albumin (BSA). Slides were incubated with primary antibodies overnight at 4 °C, followed by washing with PBS and incubation with the secondary antibodies. The following primary antibodies were used: UEB2C (Abcam, ab252940, 1:50), Nuclei were labelled blue with DAPI. The images were captured by fluorescence microscopy (Olympus).

### Cell culture

The retinoblastoma cell line Y79 was obtained from ATCC, and the cell line WERI-Rb1 was obtained from the Cell Bank/Stem Cell Bank (Chinese Academy of Sciences). The adult retinal pigment epithelium cell line ARPE-19 was obtained from the Cell Bank/Stem Cell Bank (Chinese Academy of Sciences). The cells were cultured in RPMI-1640 medium (Gibco, USA). All the media were supplemented with 10% foetal bovine serum (Gibco, USA), 1% penicillin and streptomycin, and the cells were incubated at 37 °C with 5% CO_2_.

### Western blot analysis

The antibodies used in western blot analysis were UBE2C (Abcam, ab252940, 1:1000) and GAPDH (Bioworld, MB001, 1:5000). The immunoblots were visualized with the Odyssey infrared imaging system (LI-COR).

### shRNA assay

The two shRNA sequences targeting UBE2C were cloned into the pLKO.1-puro vector (Addgene). The sequences used to target UBE2C were listed as follows: CCGGGCCTGTCCTTGTGTCGTCTTTCTCGAGAAAGACGACACAAGGACAGGCTTTTTG and CCGGTGTCTGGCGATAAAGGGATTTCTCGAGAAATCCCTTTATCGCCAGACATTTTTG.

### CCK8 assay

To determine cell viability, cells were seeded in 96-well plates at a density of 3000 cells per well. After incubation with 10 μL CCK-8 reagent (Dojindo Laboratories, Japan) per well, the absorbance was measured at a wavelength of 450 nm at the indicated time points. The data were recorded and analysed. The results were presented as the mean ± SEM.

### Plate colony formation assay

UBE2C-knockdown Y79 cells (2000 cells per well) or the Control cells were plated in 12-well plates (Poly-lysine-coated 12-well plates, WHB, China) and incubated in complete culture medium for 8 days. The colonies were stained with crystal violet and counted. The number of colonies was recorded between the groups. The colonies were washed with PBS and then fixed and stained with a 0.5% crystal violet solution. Images were captured by a scanner, and the number of colonies in each well was detected by ImageJ software.

### Soft agar colony-formation assay

A volume of 1 mL of 0.6% agar (Sigma-Aldrich, USA) in the complete medium was spread in each well of a 12-well plate; UBE2C-knockdown Y79 cells (2000 cells per well) or the Control cells were suspended in 1.0 mL of 0.3% agar complete medium and seeded into the upper layer. The cells were cultured with 300 µL of complete medium for 4 weeks. Images were captured by a camera, and the number of colonies in each well was detected by ImageJ software.

### In vivo animal model experiments

A total of 1 × 10^6^ tumour cells was implanted on the retinas through intraocular injection to establish a stable orthotopic retinoblastoma model in BALB/c nude mice (male, 4-weeks old). Mice were randomly divided into two groups: the Control group (*N* = 6 eyes) and the UBE2C-knockdown group (*N* = 6 eyes). Then, the mice were euthanized, and tumour bearing eyeballs were removed, fixed in 4% paraformaldehyde and weighted. All experimental procedures were approved by the Institutional Animal Care and Use Committee of the Ninth People’s Hospital, Shanghai Jiao Tong University School of Medicine

### Statistical analysis

All the experimental data are presented as the mean ± SEM error. For statistical analysis, GraphPad Prism 7.0 software (GraphPad Software, San Diego, CA) was used. Differences between two groups were analysed by two-tailed Student’s *t*-test while differences among multiple groups were analysed by two-way analysis of variance (ANOVA). *P* < 0.05 was considered statistically significant.

## Supplementary information

Figure legend

s-Figure 1

s-Figure 2

s-Figure 3

s-Figure 4

s-Figure 5

s-Figure 6

s-Figure 7

s-Table 1

s-Table 2

## Data Availability

All data needed to evaluate the conclusions of the paper are presented in the paper and/or Supplementary Materials. Additional data related to this paper may be requested from the authors. Single-cell RNA-seq data that support the findings of this study have been deposited in Gene Expression Omnibus (GEO) with the accession code PRJNA737188.
