# Supplementary material for: Single-cell transcriptome profiling reveals intratumoural heterogeneity and malignant progression in retinoblastoma
Source: Cell Death Dis. 2021 Nov 23;12(12):1100. doi: 10.1038/s41419-021-04390-4 (PMC8611004; doi:10.1038/s41419-021-04390-4)
Supplement: Supplementary file 1 — Figure legend [file 41419_2021_4390_MOESM1_ESM.docx]

**Supplementary Figure legends**

**Supplementary Fig. 1 Patient clinical information**

A. Photos of two patients with leucocoria.

B. Retcam photography showing the intraretinal tumours.

C. MRI showed the intraretinal tumours.

D. Histopathology revealed poorly differentiated retinoblastoma.

**Supplementary Fig. 2 Data quality control**

A. Violin plots show before and after quality control (QC).

B. The outliers.

C. Violin plots show the distribution of genes and UMIs detected per cell.

**Supplementary Fig. 3 Expression of mature cone cell marker genes in ten cell types**

A. Feature expression heatmap showing expression patterns of major retinal class markers across 10 cell clusters. In a given cell identify, the sizes of circles indicate percentage of the cells expressing each marker gene; The shades of blue indicate average expression of each gene.

B. t-SNE plots showing expression of a set of selected marker genes for c9.

**Supplementary Fig. 4** **RNA Velocity analysis showing the origin of retinoblastoma cells and inter-relationship of five main populations (c4, c5, c7, c8, c10).**

**Supplementary Fig. 5 Expression of RB1, ARR3, MYCN and ATOH7 in five states of cells**

A, B, C, D. Jitter plots showing the expression level of the RB1 (A), ARR3 (B), MYCN (C) and ATOH7 (D) changing with pseudotime, color-coded according to cell clusters (c1, c2, c3, c4, c5, c6, c7, c8, c10).

**Supplementary Fig. 6 Prognostic role of UBE2C in different tumours**

A. Heatmap of UBE2C in bulk retinoblastoma by RNA-seq.

B. Data-mining analysis of UBE2C from GEPIA. Expression profile of UBE2C in 31 different tumour types and reference non-tumour tissue.

C. The expression profiles of UBE2C in stages of eight different tumour types from GEPIA.

**Supplementary Fig. 7 The correlation between UBE2C and RB1**

A, B. UBE2C expression significantly correlated with RB1 expression in retinoblastoma (A) and neuroblastoma (B) amplification clinical tumour samples from the TCGA database.
