# Supplementary figures and images for: Single-cell transcriptome profiling reveals intratumoural heterogeneity and malignant progression in retinoblastoma
Source: Cell Death Dis. 2021 Nov 23;12(12):1100. doi: 10.1038/s41419-021-04390-4 (PMC8611004; doi:10.1038/s41419-021-04390-4)

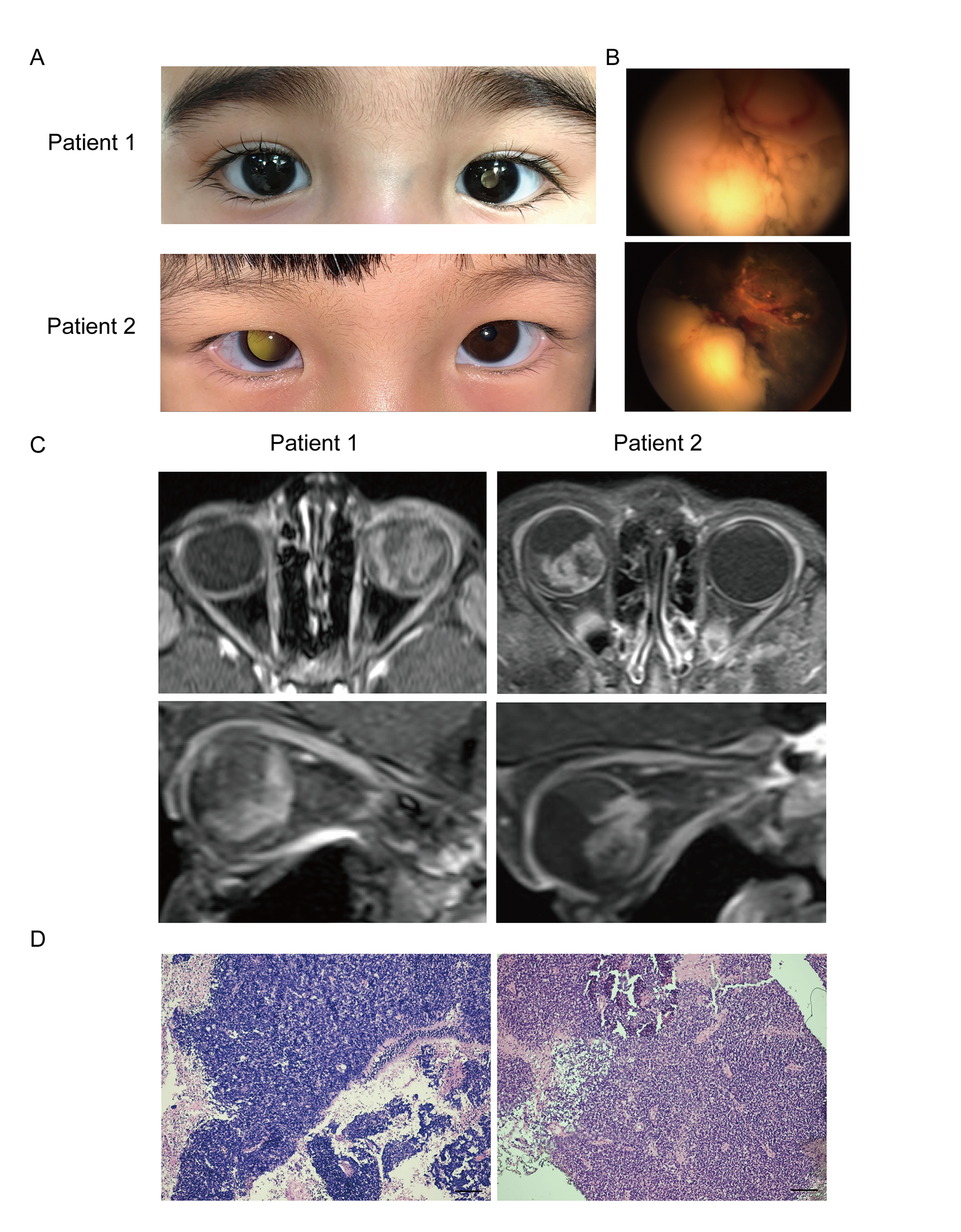

Supplement: Supplementary file 2 — s-Figure 1 [file 41419_2021_4390_MOESM2_ESM.tif]

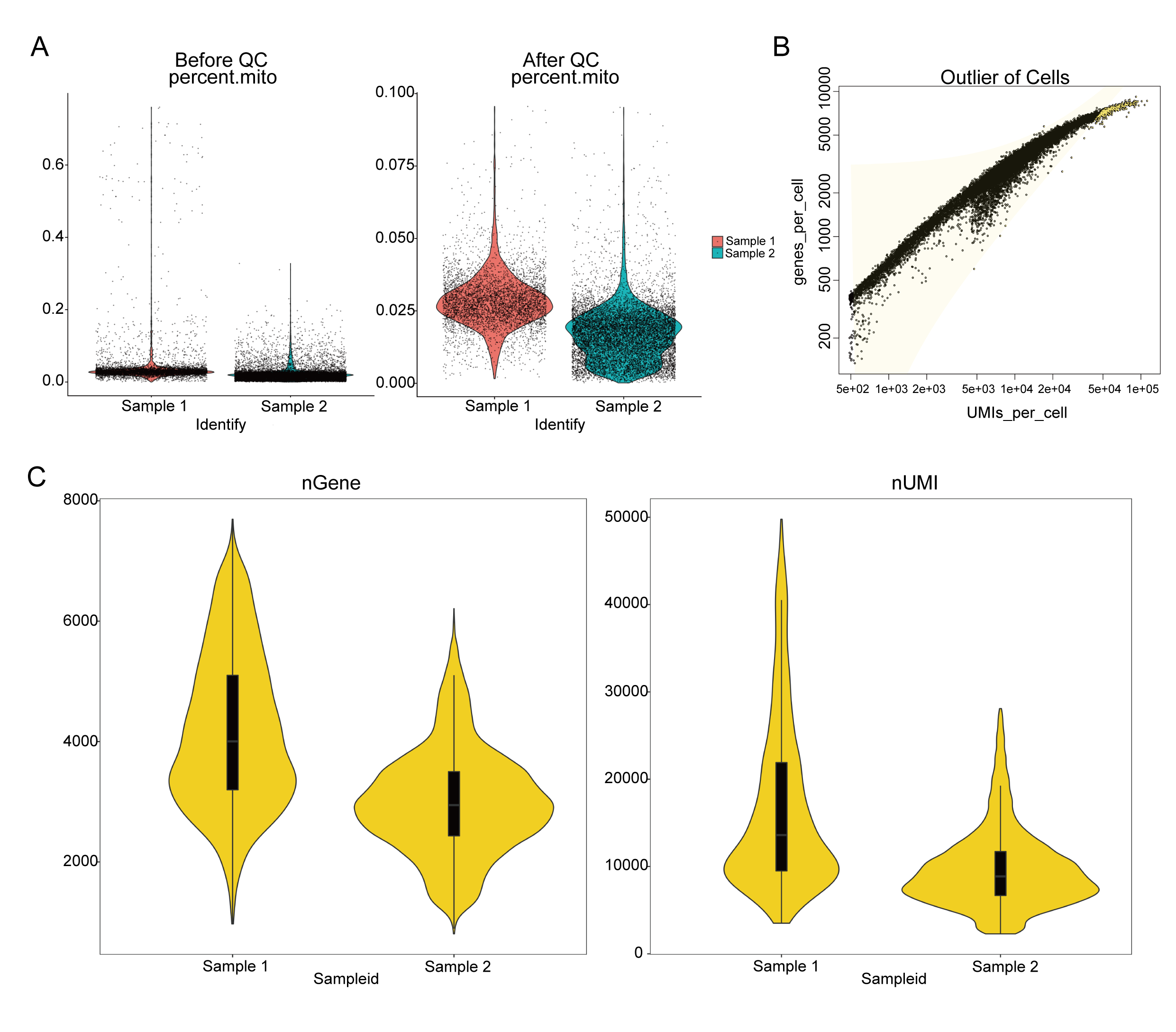

Supplement: Supplementary file 3 — s-Figure 2 [file 41419_2021_4390_MOESM3_ESM.tif]

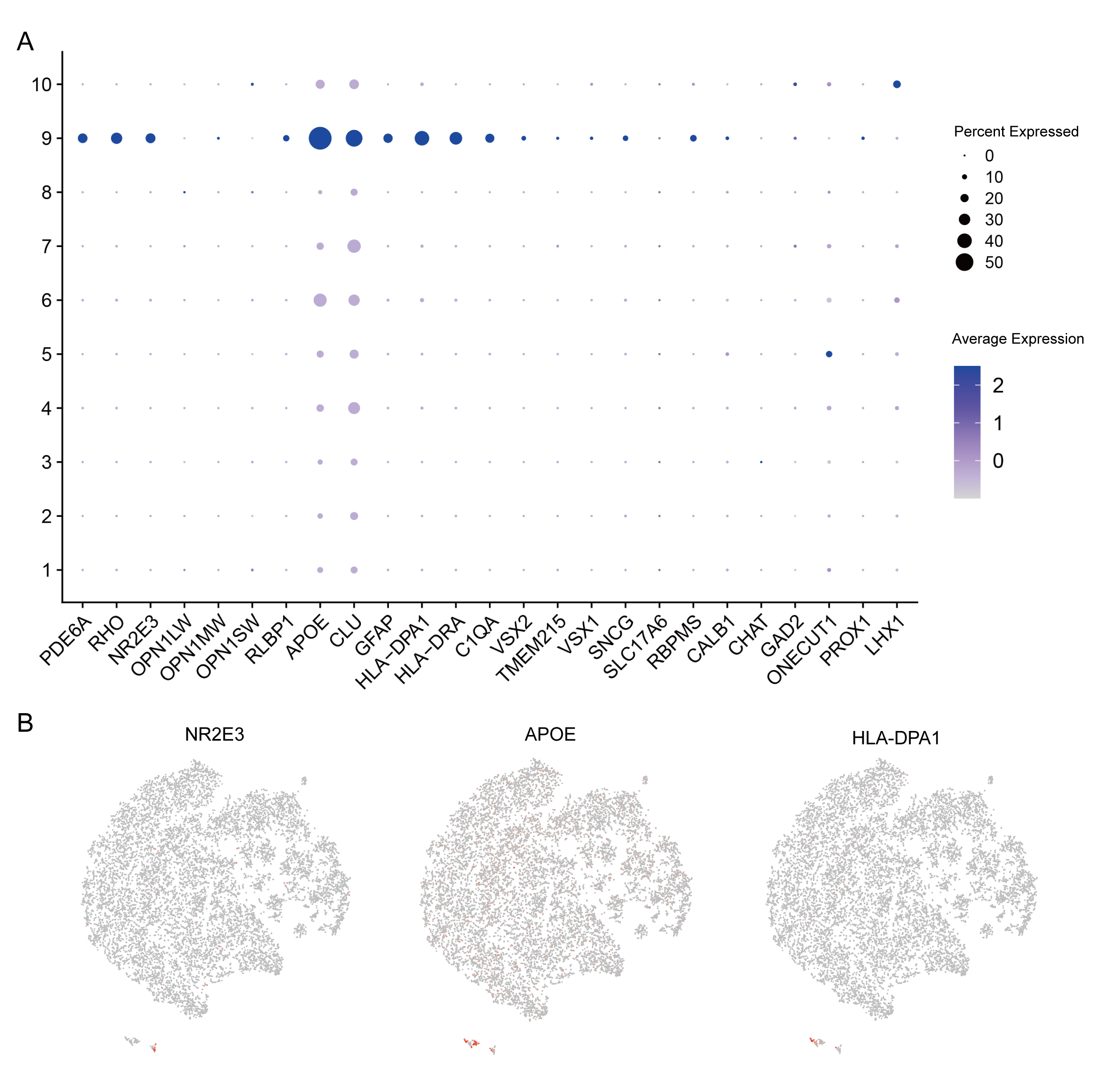

Supplement: Supplementary file 4 — s-Figure 3 [file 41419_2021_4390_MOESM4_ESM.tif]

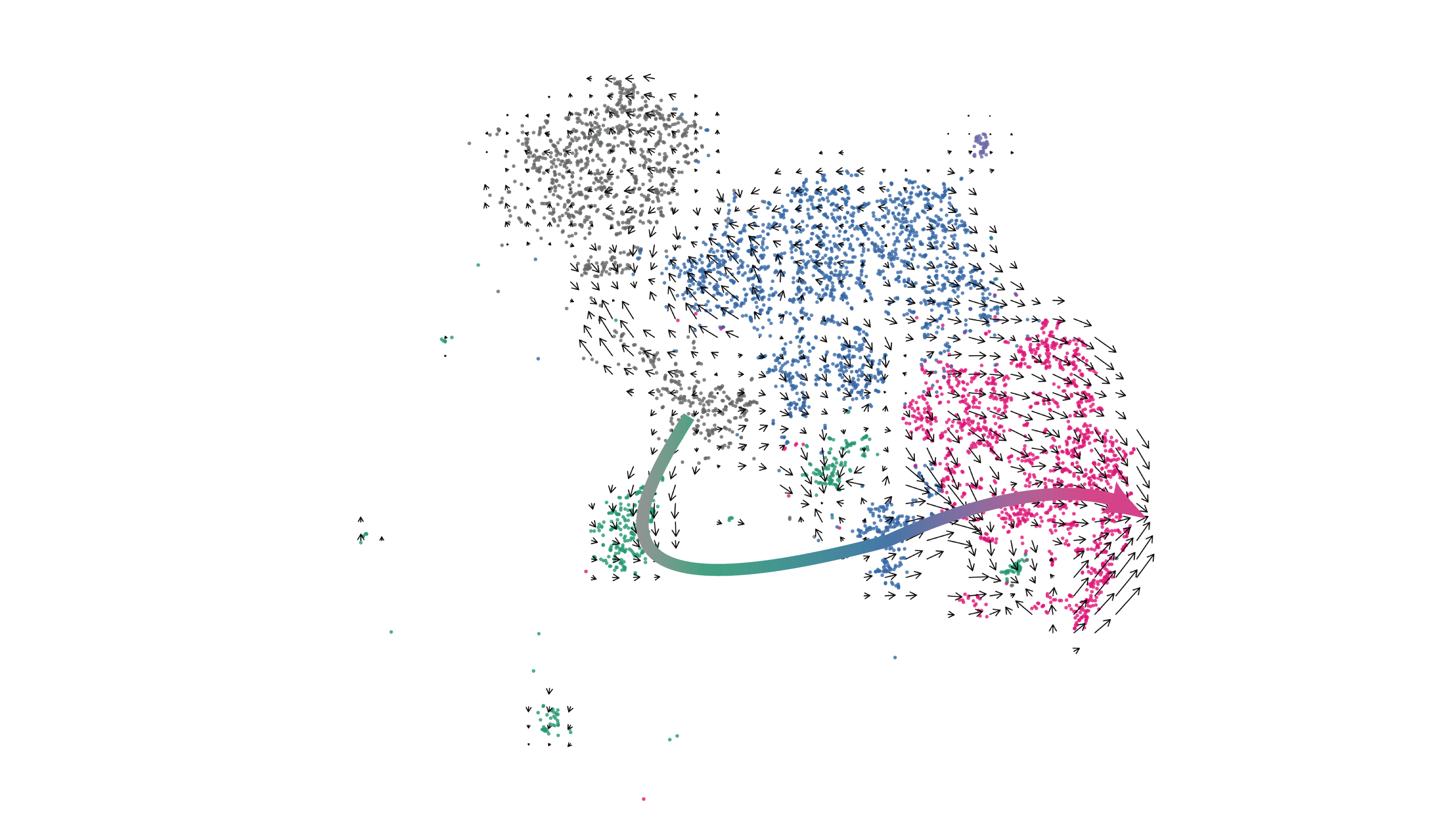

Supplement: Supplementary file 5 — s-Figure 4 [file 41419_2021_4390_MOESM5_ESM.tif]

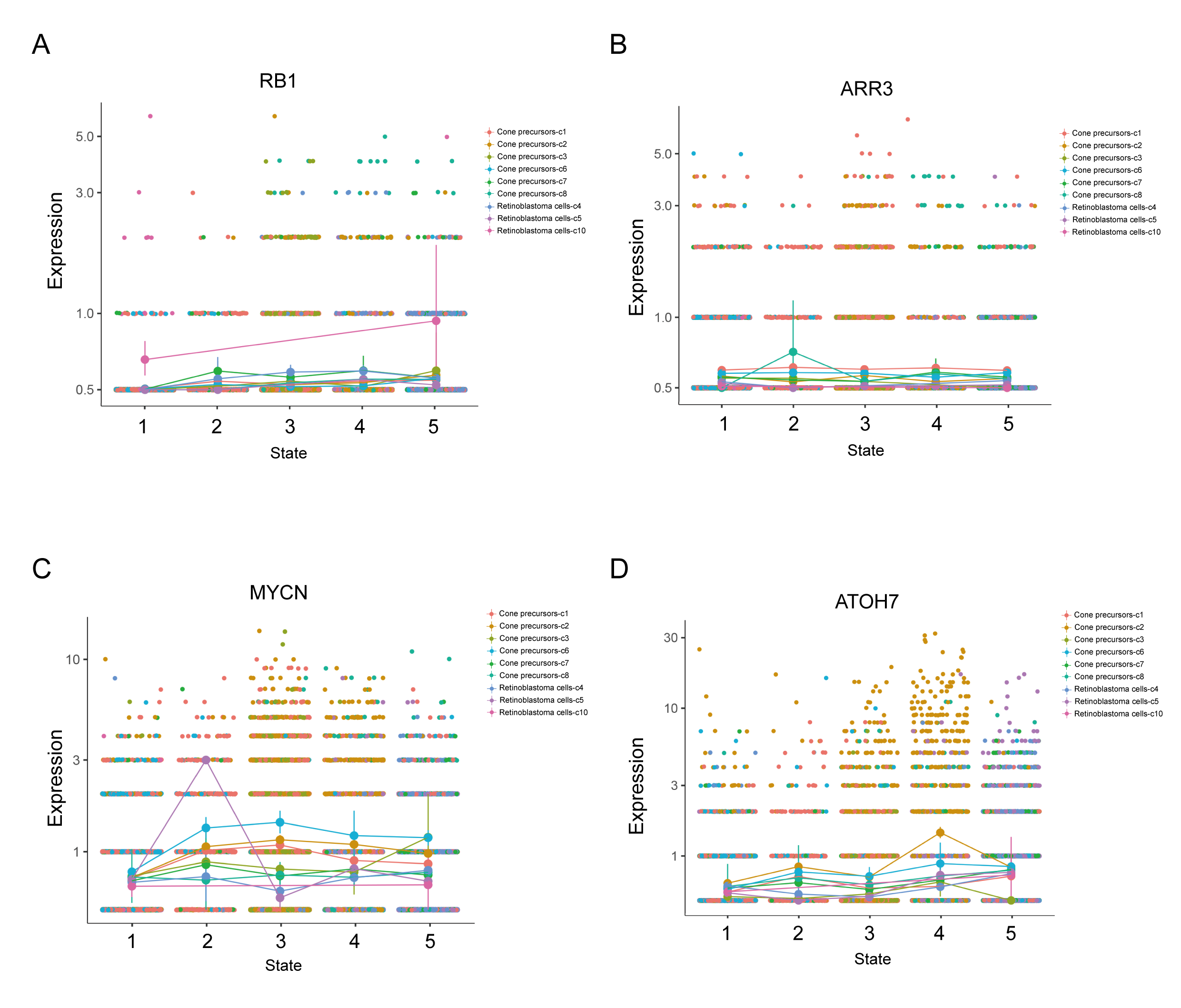

Supplement: Supplementary file 6 — s-Figure 5 [file 41419_2021_4390_MOESM6_ESM.tif]

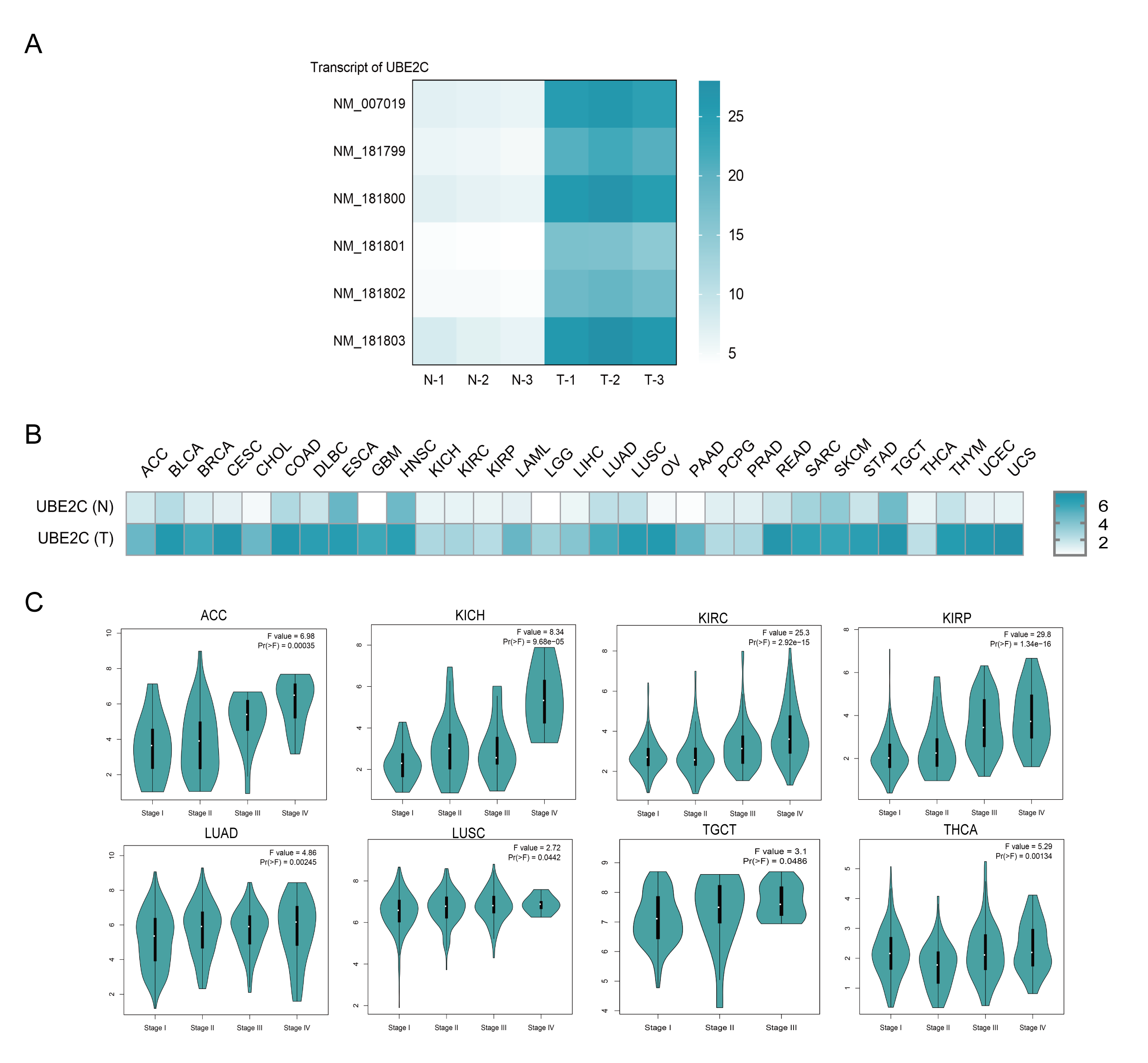

Supplement: Supplementary file 7 — s-Figure 6 [file 41419_2021_4390_MOESM7_ESM.tif]

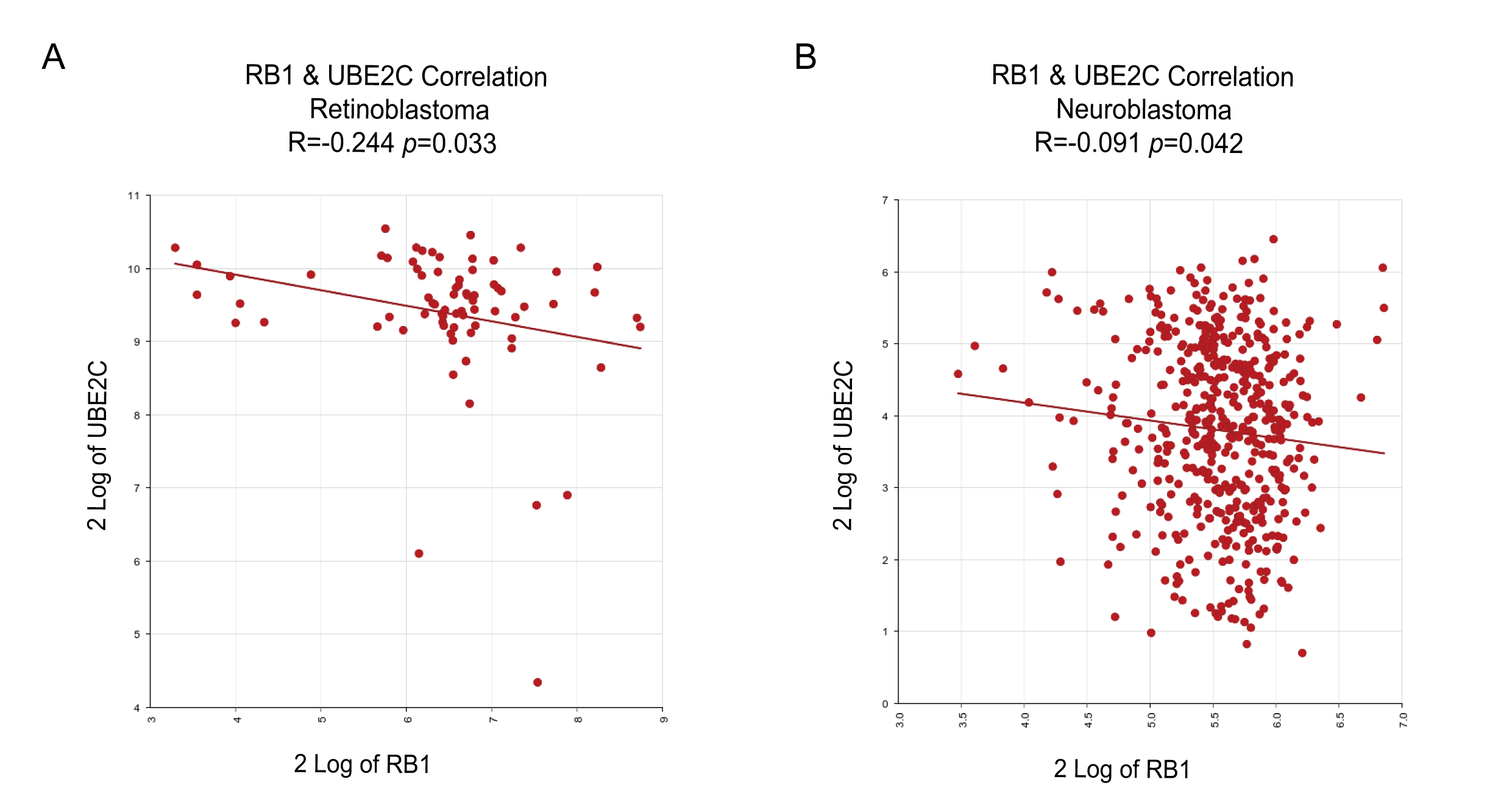

Supplement: Supplementary file 8 — s-Figure 7 [file 41419_2021_4390_MOESM8_ESM.tif]
