# Supplementary material for: Single-cell transcriptome profiling reveals intratumoural heterogeneity and malignant progression in retinoblastoma
Source: Cell Death Dis. 2021 Nov 23;12(12):1100. doi: 10.1038/s41419-021-04390-4 (PMC8611004; doi:10.1038/s41419-021-04390-4)
Supplement: Supplementary file 9 — s-Table 1 [file 41419_2021_4390_MOESM9_ESM.docx]

Supplementary Table 1 Characteristics of patients

| Sample | Age (months) | Gender | Affected side | | Family history | IIRC | Treatment |
| --- | --- | --- | --- | --- | --- | --- | --- |
| 1 | 30 | Female | | Left | No | E | Enucleation |
| 2 | 32 | Female | | Right | No | E | Enucleation |

IIRC: intraocular international retinoblastoma classification
